# Pharmacokinetic bioequivalence of the fixed-dose combination of pertuzumab and trastuzumab administered subcutaneously using a handheld syringe or an on-body delivery system

**DOI:** 10.1007/s00432-025-06228-4

**Published:** 2025-06-14

**Authors:** Chris Wynne, Bei Wang, Rong Deng, Junyi Li, Daniel Eiger, Fabiola Bene Tchaleu, Sarah Heeson, Eleonora Restuccia

**Affiliations:** 1 New Zealand Clinical Research, Christchurch, New Zealand; 2 Clinical Pharmacology, Genentech Research and Early Development, Genentech, Inc., South San Francisco, CA USA; 3 Product Development Oncology, F. Hoffmann-La Roche Ltd, Basel, Switzerland; 4 Genentech, Inc., South San Francisco, CA USA; 5 Product Development Oncology– Clinical Science Oncology, Roche Products Limited, Welwyn Garden City, UK; 6 Present Address: Current affiliation: Bristol Myers Squibb, Steinhausen, Switzerland

**Keywords:** Bioequivalence, On-body delivery system, Pharmacokinetics, Bioequivalence, Pertuzumab, Trastuzumab

## Abstract

**Purpose:**

This randomized, open-label, two-arm, parallel-group, single dose, multi-center phase I study (ClinicalTrials.gov ID, NCT05275010) investigated the comparability of the pharmacokinetics of a new formulation combining pertuzumab (P) and trastuzumab (H) in one fixed-dose combination for subcutaneous injection (FDC SC) using a proprietary on-body injector (OBI) or a handheld syringe with hypodermic needle in healthy male subjects.

**Methods:**

Healthy male subjects were randomized 1:1 to either PH FDC SC using a handheld syringe (Arm 1) or an OBI device (Arm 2). Co-primary endpoints were: (i) area under the time–concentration curve (AUC) from the start of dosing to day 63 (AUC_0–62_) of serum P, (ii) maximum serum concentration (C_max_) from start of dosing to 63 days of serum P, (iii) AUC from the start of dosing to day 63 (AUC_0–62_) of serum H, and (iv) C_max_ from start of dosing to 63 days of serum H. Safety was a key secondary endpoint. Liquid chromatography coupled to tandem mass spectrometry was used to measure pertuzumab and trastuzumab simultaneously in serum samples.

**Results:**

The obtained geometric mean ratios for C_max_ and AUC_0–62_ were within the pre-specified bioequivalence margins (0.80, 1.25) for both P and H, therefore meeting the criteria for bioequivalence. No discontinuations due to safety reasons were reported. Overall, the final safety analysis with longer follow-up was consistent with the primary analysis; there were no new or unexpected safety findings.

**Conclusion:**

This study demonstrated the feasibility of a hands-free device approach to deliver pertuzumab and trastuzumab in one fixed-dose combination for subcutaneous injection without compromising pharmacokinetics and safety.

**Supplementary Information:**

The online version contains supplementary material available at 10.1007/s00432-025-06228-4.

## Introduction

Approximately 20% of breast cancers overexpress human epidermal growth factor receptor 2 (HER2) (Tesch and Gelmon 2020). Patients with HER2-positive (HER2+) breast cancer have a poor prognosis, including a greater risk of relapse and shortened survival, compared with patients who have HER2-negative (HER2–) breast cancer (Slamon et al. 1987; Toikkanen et al. 1992; Andrulis et al. 1998; Pauletti et al. 2000; Rubin and Yarden 2001). Prognosis of patients with HER2+ breast cancer has significantly improved with the use of the monoclonal antibody (mAb) trastuzumab (H), which is capable of blocking HER2 oncogenic signaling and thereby effectively killing HER2+ cancer cells (Hudis 2007). In addition, pertuzumab (P), a second mAb that binds to a different epitope of HER2 than H, was shown to provide a complementary mechanism for disrupting HER2 signaling, resulting in enhanced anti-tumor activity (Eiger et al. 2019). Compared with single HER2 blockade, dual HER2 blockade with a combination of P and H has been shown to improve pathological complete response rates, invasive disease-free survival, and overall survival (Gianni et al. 2012; Piccart et al. 2021; Swain et al. 2020). The combination of P with H and chemotherapy has therefore become the standard of care for treating HER2+ early and metastatic breast cancer and paved the way for the development of a new product combining P and H in one fixed-dose combination (FDC) for subcutaneous (SC) injection (PH FDC SC) (Denduluri et al. 2018; Giordano et al. 2018; Cardoso et al. 2019, 2020).

PH FDC SC is a ready-to-use formulation of P and H co-formulated in a single vial for SC injection use with recombinant human PH20 hyaluronidase (rHuPH20), a permeation enhancer developed to improve dispersion of large volumes of drugs when administered subcutaneously (Tan et al. 2021; Genentech, Inc. 2020). PH FDC SC has been shown to be non-inferior to intravenous (IV) P + H from a pharmacokinetic (PK) perspective, with comparable efficacy and safety profiles (Tan et al. 2021). PH FDC SC was approved by the US Food and Drug Administration (FDA) and the European Medicines Agency (EMA) in 2020 for use in HER2+ early and metastatic breast cancer based on the pivotal phase III FeDeriCa study (NCT03493854) (Genentech, Inc. 2020; Roche Registration GmbH 2021).

The administration of PH FDC SC by lay caregivers or self-administered by patients at home using an on-body injector (OBI) could increase convenience for patients while simultaneously decreasing healthcare costs associated with repeated clinic and/or hospital visits (Bittner et al. 2018). In turn, this could also allow patients more time to engage in other activities and increase compliance of patients that live far from a healthcare facility or have difficulty travelling (Bittner et al. 2018; Viola et al. 2018). The phase II PHranceSCa study, which aimed to assess patient preference for PH administration, indicated that 85.0% of patients preferred PH FDC SC over P + H IV, which was preferred by only 13.8% of patients (O’Shaughnessy et al. 2021). PH FDC SC was found to be preferred by most patients due to a shorter administration time of 5–8 min compared with P + H IV, which can take hours (O’Shaughnessy et al. 2020). P + H IV and PH FDC SC were well tolerated. Few Grade ≥ 3 anaphylaxis/hypersensitivity events were reported with the use of PH FDC SC, with the majority of adverse events (AEs) being Grade 1 and 2 in severity. Most events typically occurred within the first 6–8 cycles when given in combination with chemotherapy (Swain et al. 2023). These results further support home administration by a healthcare professional for eligible patients appropriately selected by the physician.

To establish treatment via an OBI administration route, bioequivalence between PH FDC SC with the OBI and the currently approved handheld injection with a syringe should be demonstrated. This randomized, multicenter, open-label, two-arm, parallel-group, single-dose phase I study investigated the comparability of the PK of PH FDC SC using the proprietary on-body delivery system or a handheld syringe with hypodermic needle in healthy male subjects (ClinicalTrials.gov ID, NCT05275010). Here we report PK, safety, and device monitoring data from the primary and final analyses of this study of PH FDC SC administration via an OBI device vs. a handheld syringe.

## Methods

### Subjects and study design

Eligible subjects were healthy males aged between 18 and 45 years. Subjects that received previous anti-cancer treatment, including P, H, anthracyclines, or any cardiotoxic drugs were excluded from the study. Eligible subjects did not have any significant comorbidities; they were required to have a body mass index between 18 and 32 kg/m^2^ and baseline left ventricular ejection fraction (LVEF) ≥ 55% measured by echocardiogram. They were to have no history or family history of any clinically significant and clinically relevant hypersensitivity, allergy, or cardiac condition and were not eligible if they had systolic blood pressure ≥ 140 mm Hg or < 90 mm Hg or diastolic blood pressure > 90 mm Hg or < 50 mm Hg. Clinically significant abnormalities in laboratory test results were also an exclusion criterion, along with positive test results for hepatitis B virus, hepatitis C virus, or human immunodeficiency virus 1 or 2. Subjects with documented use of prohibited medications, including non-prescription medications, nutraceuticals, nutritional supplements, or any herbal remedies taken within 10 days or 5 times the elimination half-life (whichever was longer) prior to randomization into the study were excluded.

In this manuscript, the primary and secondary PK objectives, safety and device monitoring objectives are reported based on the primary analysis (clinical cut-off date [CCOD] May 14, 2023). Additional safety and exploratory objectives are reported based on the final analysis (CCOD October 3, 2023).

The protocol, protocol amendments, informed consent form, investigator brochure, and other relevant documents (e.g., advertisements) were submitted to an independent review board or ethics committee at each study site. The study was assigned number 2022-08-803 by the Australian Human Research Ethics Committee (HREC - Bellberry) and number 11,370 by the New Zealand Health and Disability Ethics Committee (HDEC). This study was conducted in accordance with the protocol and with the following: (i) consensus ethical principles derived from international guidelines including the Declaration of Helsinki and Council for International Organizations of Medical Sciences International Ethical Guidelines, (ii) applicable ICH Good Clinical Practice Guidelines, and (iii) applicable laws and regulations.

### Procedures and assessments

Healthy male subjects were randomized 1:1 to either PH FDC SC (pertuzumab 600 mg trastuzumab 600 mg/20,000 U rHuPH20) using a handheld syringe (Arm 1) or an OBI device (Arm 2).

Pain intensity scores were assessed using the Visual Analog Scale (VAS) on a line measuring between 0 mm (‘no pain’) and 100 mm (‘unbearable pain’). Site staff and subjects in the OBI arm completed the device monitoring questionnaire. The questionnaire assessed the following criteria, which were each rated on a three-point scale as “good”, “acceptable”, or “poor”: (i) ease of OBI attachment, attachment during the injection and ease of OBI removal, (ii) overall wearing comfort (subject assessed), and (iii) overall clarity of handling instructions. Assessment of skin irritation and sensitization reactions (dermal or other effects) at the site of the injection, before and after injection, were assessed by the study staff using the device monitoring questionnaire. These were assessed on a scale of 0 to 7.

### Endpoints

The objective of the study was to demonstrate comparability of single-dose AUC and C_max_ from start of dosing to day 63 for P and H within PH FDC SC administered by an OBI device vs. a hand-held syringe with a hypodermic needle. Co-primary endpoints were: (i) AUC from the start of dosing to day 63 (AUC_0–62_) of serum P, (ii) C_max_ from start of dosing to 63 days of serum P, (iii) AUC from the start of dosing to day 63 (AUC_0–62_) of serum H, and (iv) C_max_ from start of dosing to 63 days of serum H. Secondary endpoints included observed serum day 22 and day 63 concentration values for H and P, additional PK endpoints for further characterization, safety, and device monitoring.

### Pharmacokinetics sampling and bioanalytical assessments

Serum samples for measuring P and H concentrations were collected at pre-dose (0 h) and 2, 6, and 12 h post-dose on day 1, and post-dose on days 2, 3, 5, 7, 9, 11, 15, 22, 35, 49, and 63. A validated duplex hybrid immunoaffinity capture liquid chromatography tandem mass spectrometry assay (LC-MS/MS; Shimadzu LC system [Shimadzu Scientific Instruments, Columbia, MD, USA] in tandem with an API 6500 MS [Sciex, Framingham, MA, USA]) was used to simultaneously measure the concentration of both P and H in serum samples. The concentration of the lower limit of quantification for both analytes was 100 ng/mL.

Briefly, the assay used an affinity capture approach using streptavidin magnetic beads coupled with biotinylated recombinant HER2 extracellular domain to enrich P and H (both immunoglobulin molecules) simultaneously from human serum (Nijem et al. 2025). The bound proteins were subjected to “on-bead” proteolysis with trypsin, following standard protein denaturation, reduction, and alkylation processing steps to generate unique characteristic peptides. Prior to digestion completion, corresponding stable isotope-labeled peptide internal standards were added. Characteristic signature peptides were then quantified as surrogates of the total antibody concentration originating from P and H by LC-MS/MS (i.e., multiple reaction monitoring). The dynamic concentration range of the method is 100–25,000 ng/mL.

There was a total of 44 batches for PK analysis, and the criteria for acceptability were as follows. For a batch run to be acceptable, at least 75% of the total number of calibration standards or at least 6 calibration standards in the calibration range, including the lower limit of quantification and upper limit of quantification, must not have deviated by greater than ± 20.0% (± 25.0% at the method lower limit of quantification concentration) from their nominal values.

### Statistical analysis

Approximately 144 subjects were planned for enrollment, assuming a drop-out rate of 10%. A total of 130 healthy subjects with fully evaluable PK profiles were required to show comparability of area under the time–concentration curve from the beginning of dosing to 63 days (AUC_0–62_) and C_max_ values between the PH FDC SC administration via a hand-held syringe (reference) and the OBI device (test) with 80% power at a 5% significance level, assuming a between-subject coefficient of variation of 40% and a “test” to “reference” ratio of 0.95. The intention-to-treat (ITT) population was defined as all randomized subjects, whether they received the assigned treatment or not. The safety analysis population included all subjects who received a dose of study treatment. The per protocol PK analysis population for P C_max_, P AUC_0–62_, H C_max_, and H AUC_0–62_, included all randomized subjects who were dosed and adhered to the pre-specified protocol criteria. The main analytical approach for the primary endpoints was assessment of the bioequivalence of the two administration methods by a two one-sided testing procedure. The geometric mean ratio (GMR) of administration by the OBI relative to administration by handheld syringe was estimated together with the two-sided 90% confidence interval (CI) based on the log-transformed AUC_0–62_ and C_max_ values. The null hypothesis was to be rejected, and bioequivalence concluded, if the bounds of the 90% CI of the GMR of AUC_0–62_ and C_max_ values for P and H administration by the OBI relative to the administration by the handheld syringe with hypodermic needle were entirely contained within the standard bioequivalence margins (0.80; 1.25) for all co-primary endpoints. Bioequivalence between the two administration methods was to be concluded only if bioequivalence was established for both P and H AUC_0–62_ and C_max_. The GMR together with the two-sided 90% CI were estimated using analysis of variance method with log-transformed AUC_0–62_ and C_max_ values as the dependent variables and study arm as a covariate for P and H.

Phoenix WinNonlin™ (Certara, Radnor, PA, USA) was used for non-compartmental analyses; SAS (SAS Institute, Cary, NC, USA), for statistical analyses.

## Results

### Subjects and dose administration

**Fig. 1:**
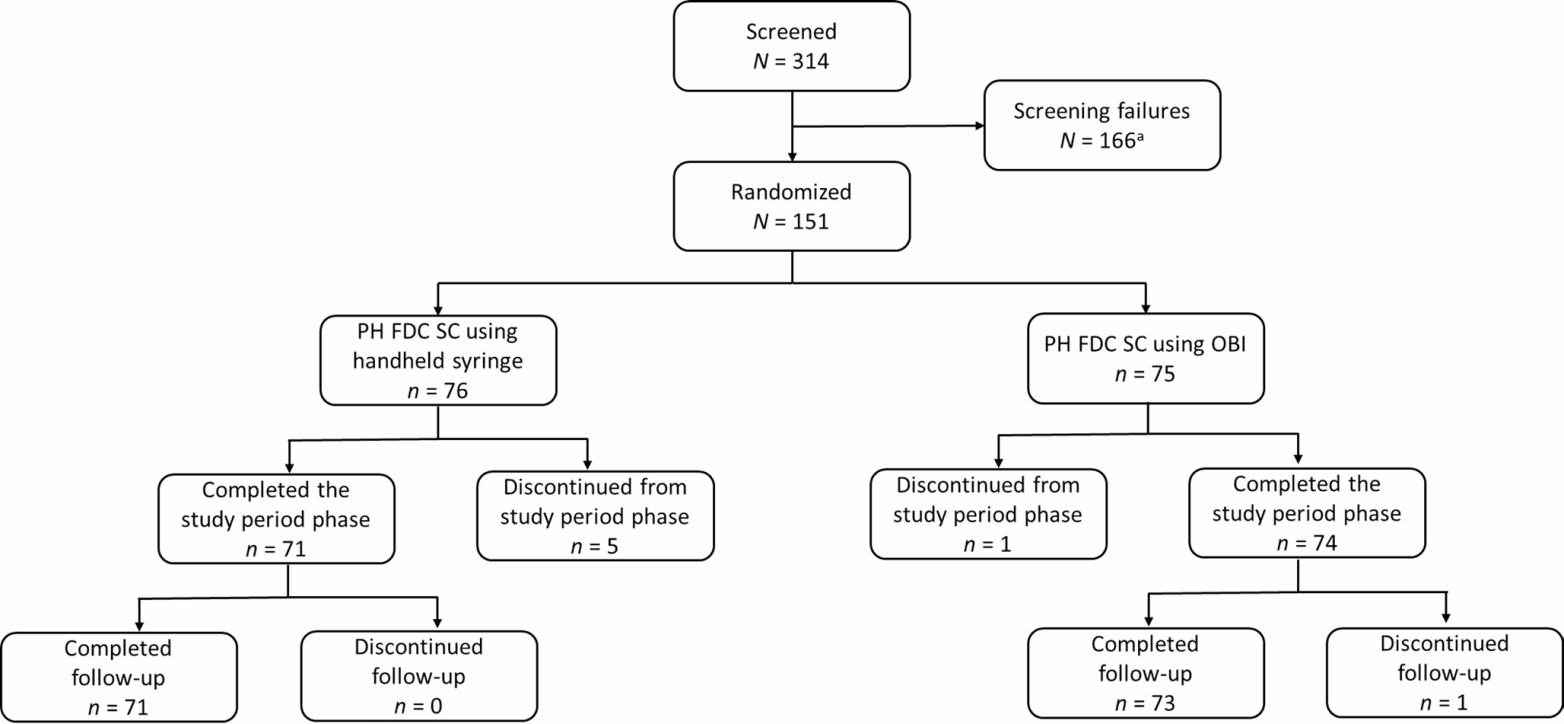
Subject disposition (primary and final analysis). ^a^Five subjects were re-screened, three of whom were subsequently enrolled. *FDC* fixed-dose combination; *H* trastuzumab; *OBI* on-body injector; *P* pertuzumab; *SC* subcutaneous. The one subject in the PH FDC SC using OBI arm discontinued follow-up for non-safety reasons (loss to follow-up)

One-hundred-and-fifty-one male subjects were enrolled in sites in Australia and New Zealand starting on May 30, 2022, with 76 randomized to the PH FDC SC arm and 75 to the PH FDC OBI arm. Analysis populations are shown in Supplementary Table 1. The last visit occurred on October 3, 2023. Baseline demographic characteristics in the ITT population are shown in Table 1. The median duration of the injection was similar in both arms (PH FDC SC arm: 5.0 min vs. PH FDC OBI arm: 6.0 min). In both arms, > 90% of subjects completed the study and follow-up period. As of the CCOD of May 14, 2023 for the primary analysis, 25 (35.2%) subjects had completed the follow-up period in the PH FDC SC arm and 29 (39.2%) in the PH FDC SC OBI arm. As of the CCOD of October 3, 2023 for the final analysis, 71 (93.4%) subjects in the PH FDC SC arm and 73 (97.3%) in the PH FDC SC OBI arm completed follow-up (Fig. 1). The most common reason for subjects discontinuing the study in both arms was unrelated to safety. In the PH FDC SC arm, out of the five subjects who discontinued, four did so before dosing on day 1 due to two adverse events (COVID-19 infection and upper respiratory tract infection), high ALT value, and ECG abnormalities. One subject withdrew from the study on day 29. In the PH FDC SC OBI arm, one subject discontinued before dosing on day 1 due to an ECG reading of QTcF > 30 ms from baseline. These subjects discontinued the study prior to treatment.

**Table 1:** Demographics and baseline characteristics: Intention-to-treat population

	PH FDC SC using handheld syringe (*n* = 76)	PH FDC SC using OBI (*n* = 75)
Age (years)		
Median (range)	29 (18.0–46.0)	29 (18.0–45.0)
Age group, *n* (%)^a^		
< 35	56 (73.7)	58 (77.3)
35–39	9 (11.8)	8 (10.7)
40–45	10 (13.2)	9 (12.0)
≥ 46^b^	1 (1.3)	0 (0.0)
Sex, *n* (%)		
Male	76 (100.0)	75 (100.0)
Ethnicity, *n* (%)		
Hispanic or Latino	7 (9.2)	8 (10.7)
Not Hispanic or Latino	68 (89.5)	65 (86.7)
Not reported	0 (0.0)	1 (1.3)
Unknown	1 (1.3)	1 (1.3)
Race, *n* (%)		
White	44 (57.9)	45 (60.0)
Asian	15 (19.7)	13 (17.3)
Native Hawaiian or Other Pacific Islander	10 (13.2)	7 (9.3)
Multiple	4 (5.3)	3 (4.0)
Not reported	2 (2.6)	1 (1.3)
Unknown	1 (1.3)	3 (4.0)
American Indian or Alaska Native	0 (0.0)	3 (4.0)
Weight (kg) at baseline (eCRF)		
Median (range)	82.5 (54.2–105.6)	80.4 (56.9–113.0)
BMI (kg/m^2^)		
Median	25.42	26.17
Range	18.4–32.0	18.6–31.8
Smoking status, *n* (%)		
Ever smoked	31 (40.8)	29 (38.7)
Never smoked	45 (59.2)	46 (61.3)
Male fertility status, *n* (%)^c^		
Yes	68 (89.5)	73 (97.3)
No	8 (10.5)	2 (2.7)
Alcohol test at baseline, *n* (%)		
Negative	76 (100.0)	75 (100.0)

*BMI* body mass index; *eCRF* electronic case report form; *OBI* on-body injector; *PH FDC SC* pertuzumab and trastuzumab in one fixed-dose combination for subcutaneous injection

^a^Additionally, one syringe-arm subject was categorized as ‘≥ 46’ based on study entry data is presented in the output although all subjects ≤ 45 years of age at study entry. This is because only the year of birth is collected and ‘15JUN’ inputted for derivation of a subject’s age

^b^One subject was mis-randomized in the stratum ‘≤ 75 kg’ for the stratification factor ‘Weight subgroup at Baseline’

^c^Male fertility status was determined to be “No” if the subject had been surgically sterilized or were congenitally sterilized. All other subjects were classed as being fertile/”Yes.”

### Pharmacokinetics

**Fig. 2:**
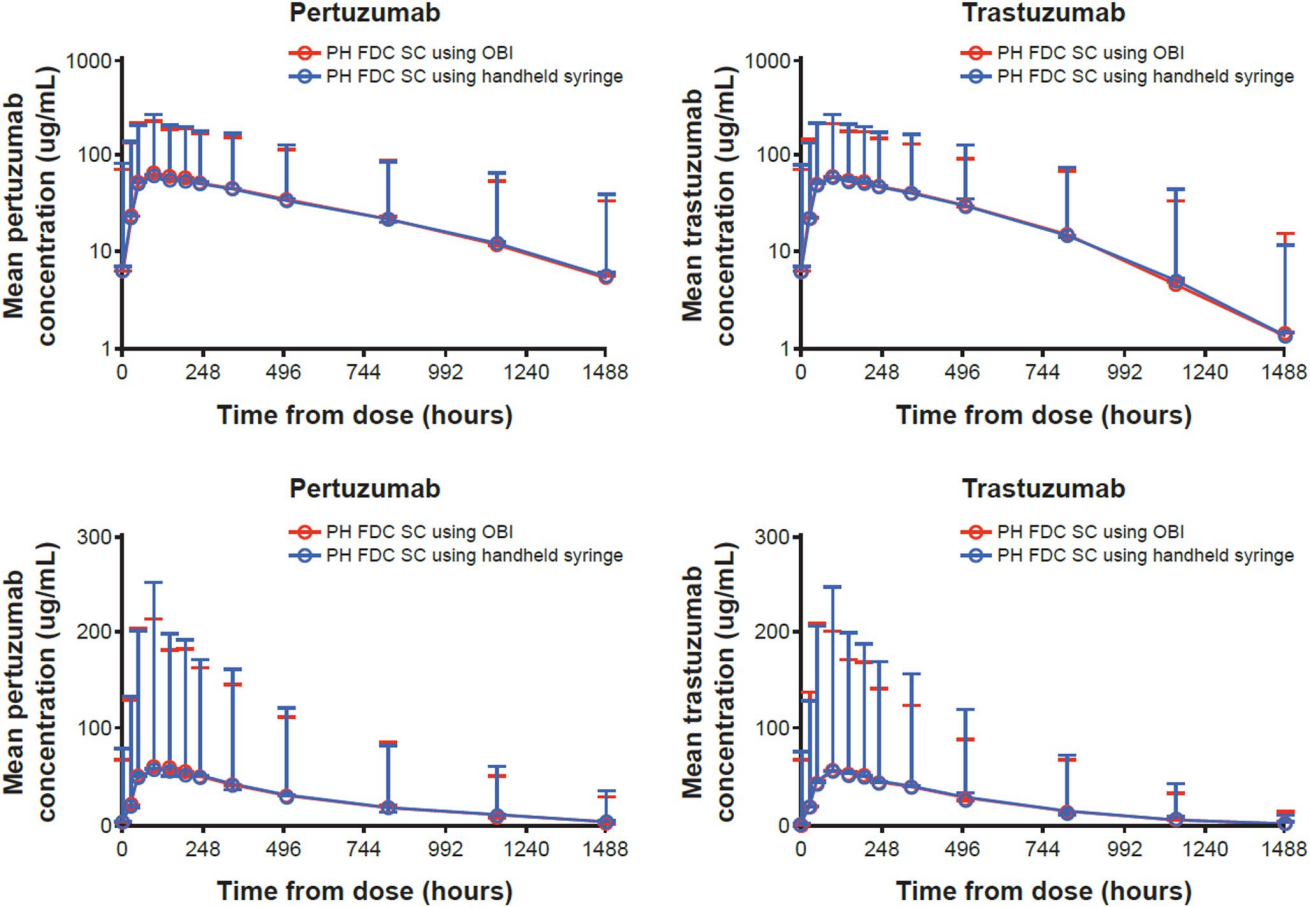
Mean (± SD) serum concentration–time plots by treatment arm (log and linear scales). *FDC* fixed-dose combination; *H* trastuzumab; *OBI* on-body injector; *P* pertuzumab; *SC* subcutaneous; *SD* standard deviation

PK summary statistics for data from the primary analysis are shown in Table 2. For P AUC_0–62_, the GMR was 1.00 (two-sided 90% CI: 0.93, 1.08). For P C_max_, the GMR was 1.04 (two-sided 90% CI: 0.96, 1.12). For H AUC_0–62_, the GMR was 1.00 (two-sided 90% CI: 0.93, 1.09). For H C_max_, the GMR was 1.04 (two-sided 90% CI: 0.95, 1.13). The resulting GMRs 90% CI were within the pre-specified bioequivalence margins (0.80, 1.25), therefore meeting the criteria for comparability. Mean (SD) serum P + H concentration profiles are included in Fig. 2.

**Table 2:** Pharmacokinetic summary statistics (primary analysis): PK evaluable population

	PH FDC SC using handheld syringe (*n* = 68)	PH FDC SC using OBI (*n* = 73)
AUC_0–62_ for pertuzumab		
Arithmetic mean (%CV), µg∙day/mL	1,738.7 (27.5)	1,722.3 (23.2) (23.2)
GMR (90% CI)	1.00 (0.93–1.08)	
C_max_ for pertuzumab		
Arithmetic mean (%CV), µg/mL	66.4 (32.2)	67.9 (24.7)
GMR (90% CI)	1.04 (0.96–1.12)	
AUC_0–62_ for trastuzumab		
Arithmetic mean (%CV), µg∙day/mL	1,413.2 (31.4)	1,385.5 (23.6)
GMR (90% CI)	1.00 (0.93–1.09)	
C_max_ for trastuzumab		
Arithmetic mean (%CV), µg/mL	63.2 (33.8)	64.0 (25.2)
GMR (90% CI)	1.04 (0.95–1.13)	

*AUC* area under the time–concentration curve; *CI* confidence interval; *C*_*max*_ maximum serum concentration; *CV* coefficient of variation; *GMR* geometric mean ratio; *OBI* on-body injector; *PH FDC SC* pertuzumab and trastuzumab in one fixed-dose combination for subcutaneous injection

### Safety analysis (primary analysis)

AEs were graded according to NCI CTCAE v5.0 and a summary is presented in Table 3. Most AEs were low grade, occurring at comparable frequency between arms; the most common related AEs occurring in at least 10% of subjects in each arm of the safety population are presented in Table 4. One subject in the syringe arm experienced a related Grade 3–4 AE (transaminases increased), and there were no Grade 3–4-related AEs in the OBI arm. There was one serious AE in the syringe arm (and none in the OBI arm), which was unrelated to study treatment (motorcycle accident). No AEs of special interest (asymptomatic decline in LVEF requiring treatment or leading to discontinuation of HER2-targeted therapy, potential cases of drug-induced liver injury, and suspected transmission of an infectious agent by study treatment) were reported. AEs of injection-related reactions occurred in 48 subjects (66.7%) in the syringe arm and in 35 subjects (47.3%) in the OBI arm. No AEs suggesting anaphylactic reactions were observed in either arm.

**Table 3:** Safety summary and adverse events to monitor in the safety population (primary analysis)

Subjects, *n* (%)	PH FDC SC using handheld syringe (*n* = 72)	PH FDC SC using OBI (*n* = 74)
**Summary**		
Overall total number of AEs (all grades)	261	234
Total subjects with ≥ 1 AE (all grades)	70 (97.2)	71 (95.9)
Grade 3–4	1 (1.4)	0 (0.0)
Grade 5	0 (0.0)	0 (0.0)
Serious AE	1 (1.4)	0 (0.0)
Related serious AE (all grades)	0 (0.0)	0 (0.0)
AE leading to drug interruption	0 (0.0)	0 (0.0)
Treatment-related AE	63 (87.5)	61 (82.4)
Device-related AE	0 (0.0)	0 (0.0)
**AEs to monitor**		
All	63 (87.5)	59 (79.7)
Infusion-/administration-related reactions within 24 h (all grades)	62 (86.1)	55 (74.3)
Grade ≥ 3	0 (0.0)	0 (0.0)
Anaphylaxis and hypersensitivity (all grades)	48 (66.7)	35 (47.3)
Grade ≥ 3	0 (0.0)	0 (0.0)
Diarrhea (all grades)	17 (23.6)	9 (12.2)
Grade ≥ 3	0 (0.0)	0 (0.0)
Neutropenia/febrile neutropenia (all grades)	0 (0.0)	1 (1.4)
Grade ≥ 3	0 (0.0)	0 (0.0)

*AE* adverse event; *BMI* body mass index; *OBI* on-body injector; *PH FDC SC* pertuzumab and trastuzumab in one fixed-dose combination for subcutaneous injection

**Table 4:** Summary of adverse events related to the study treatment in ≥ 10% of subjects in the safety population (primary analysis)

MedDRA System Organ Class MedDRA preferred term	PH FDC SC using handheld syringe (*n* = 72)	PH FDC SC using OBI (*n* = 74)
Injury, poisoning, and procedural complications		
Injection-related reaction, *n* (%)	48 (66.7)	35 (47.3)
General disorders and administration site conditions		
Injection site reaction, *n* (%)	17 (23.6)	18 (24.3)
Nervous system disorders		
Headache, *n* (%)	8 (11.1)	11 (14.9)

For frequency counts by preferred term, multiple occurrences of the same AE in an individual are counted only once

*AE* adverse event; *MedDRA* Medical Dictionary for Regulatory Activities; *OBI* on-body injector; *PH FDC SC* pertuzumab and trastuzumab in one fixed-dose combination for subcutaneous injection

### Safety analysis (final analysis)

Overall, the final safety analysis results were consistent with the primary analysis results; there were no new or unexpected safety findings. At the time of the final analysis, the number of subjects with at least one AE remained the same; however, three and two additional AEs were reported in the syringe and OBI arms, respectively. In the syringe arm, the three additional AEs were: (i) chlamydial infection, (ii) nail bed infection, and (iii) dermatitis acneiform. In the OBI arm, two additional AEs of coronavirus disease 2019 (COVID-19) and chlamydial infection were reported. One additional Grade 1 AE of injection-related reaction in the OBI arm was reported, which was initially reported during the primary analysis as an AE of myalgia and subsequently updated.

### Device monitoring (primary analysis)

No AEs related to the OBI device itself were reported. A sensation of mild pain (median < 10 mm) throughout and after administration in both arms was reported and was comparable across subjects in both arms (*n* = 72 in the PH FDC SC using a handheld syringe arm; *n* = 74 in the PH FDC SC using an OBI device arm). No subjects had any dermal effects or other effects prior to injection using the OBI device. In total, 84% of subjects had no evidence of dermal effects or irritation after injection, while 16% experienced minimal erythema using the OBI device. One subject using the OBI device experienced an effect classified as “slight glazed appearance after injection”. No malfunctions or other issues with the cartridge or device itself were reported. Overall, most site staff indicated that the device was easy to use with good adhesion. Subjects indicated that the device had good comfort. Summaries of the Visual Analogue Scale (assessment of pain), skin irritation and sensitization reactions by timepoint, and a summary of the OBI device assessment are shown in Supplemental Tables 2–4.

## Discussion

In this phase I study, the bioequivalence of PKs of the fixed-dose combination of P and H administered subcutaneously (PH FDC SC) using a handheld syringe or on-body delivery system was evaluated in healthy male subjects. The study met its primary objective; the evaluated PK metrics of PH FDC SC administered via an OBI device were equivalent to those from administration via syringe and needle. Although the PK sample collection window up to 63 days captured only up to approximately 90% of P elimination, given the device impact is mainly on absorption kinetics, the selected time period sufficiently captured this process. Safety was comparable in both arms with no new safety signals reported. Infusion-/administration-related reactions occurred at a relatively higher incidence than anticipated in both study arms, with no particular reason for a higher incidence in either arm, although both arms were comparable to the phase I dose-finding study (Kirschbrown et al. 2018). In the current study, there were no AEs suggesting anaphylaxis reactions in either arm. The pain score experienced by most subjects was mild and comparable in both arms throughout and after administration. No AEs/serious AEs related to the device itself were reported. Minimal skin irritation/sensitization caused by the adhesive on the device was observed. The OBI device was found to be generally easy to use; most site staff reported good device performance assessment scores. No issues with the cartridge or device itself or malfunctions were reported.

Similar results to those observed in this study were obtained in a bioequivalence PK study of SC H administered via handheld syringe or proprietary single-use injection device (SID) in healthy male subjects (Wynne et al. 2013). In the bioequivalence study, the GMR was 1.01 (90% CI: 0.96, 1.07) for AUC_0–21_ days and 1.02 (90% CI: 0.96, 1.10) for C_max_, which fell within the pre-specified bioequivalence range (0.80; 1.25) (Wynne et al. 2013). In addition, no SID quality issues or failures occurred, and AEs were mostly mild, with no deaths, AE-related withdrawals, or life-threatening, cardiac, or serious events reported (Wynne et al. 2013). According to guidance from past examples, the parallel, single-dose bioequivalence studies have efficiently served as the bridge between auto-injectors and pre-filled syringes for both single-agent H and combination of P + H, given the underlying principles (Li et al. 2024; Hu et al. 2020).

One limitation to the study was that since it was designed to evaluate the device in healthy male subjects, the OBI device was not tested in its target population (i.e., patients with HER2+ breast cancer). Therefore, neither data on the ease of use of the OBI for self-administration of PH FDC SC or administration by a lay caregiver, nor data on home-based administration of PH FDC SC, were generated. Nonetheless, no PK difference between healthy male subjects and HER2+ breast cancer patients should be expected (Kirschbrown et al. 2018). This is in line with the nature of this phase I bioequivalence study and further study data from ongoing studies could address these questions. For example, an expanded access study provided at-home administration of a PH FDC SC by a healthcare provider for patients with HER2+ breast cancer during the COVID-19 pandemic (NCT04395508), and a phase IIIb study is evaluating patient preference of PH FDC SC administration in a home vs. hospital setting during the crossover period of adjuvant treatment for early or locally advanced/inflammatory HER2+ breast cancer (NCT05415215, ProHer).

Automating a 5–8 min manual injection with an OBI could reduce healthcare providers’ workloads by minimizing hands-on time and ensuring consistent drug delivery. While our study did not collect data on this aspect, its impact on clinical workflow remains relevant and should be considered in future research.

## Conclusions

This study indicates that adoption of a hands-free device approach to deliver PH FDC SC without compromising PK and safety is possible. Overall, these findings suggest that a new horizon for future developmental strategies and device options in the field of SC drug delivery can be pursued to facilitate caregiver or patient self-administration at home.

## Electronic Supplementary Material

Below is the link to the electronic supplementary material.

Supplementary Material 1

## Data Availability

Qualified researchers may request access to individual subject-level data through the clinical study data request platform: https://vivli.org/. Further details on Roche’s criteria for eligible studies are available here: https://vivli.org/members/ourmembers/. For further details on Roche’s Global Policy on the Sharing of Clinical Information and how to request access to related clinical study documents, see here: https://www.roche.com/research_and_development/who_we_are_how_we_work/clinical_trials/our_commitment_to_data_sharing.htm.
