# Supplementary material for: Pharmacokinetic bioequivalence of the fixed-dose combination of pertuzumab and trastuzumab administered subcutaneously using a handheld syringe or an on-body delivery system
Source: J Cancer Res Clin Oncol. 2025 Jun 14;151(6):188. doi: 10.1007/s00432-025-06228-4 (PMC12165970; doi:10.1007/s00432-025-06228-4)
Supplement: Supplementary file 1 — Supplementary Material 1 [file 432_2025_6228_MOESM1_ESM.docx]

**Supplemental Table 1** Analysis populations

| **Analysis populations and reasons  for exclusions** | **PH FDC SC**  **using handheld syringe**  **(*n* = 76)** | **PH FDC SC**  **using OBI**  **(*n* = 75)** |
| --- | --- | --- |
| Randomized subjects | 76 | 75 |
| ITT population^a^ | 76 | 75 |
| Excluded from ITT population | 0 | 0 |
| Safety analysis population^b^ | 72 | 74 |
| Excluded as subject did not receive at least one dose of study medication | 4 | 1 |
| PAP^c^ | 71 | 74 |
| Excluded from PAP | 1 | 0 |
| Reasons for exclusion: | | |
| A BMI outside the range 18 and 32 kg/m^2^ | 0 | 0 |
| Use of prohibited medications including  non- prescription medications | 1 | 0 |
| Concomitant SC, IV, or any parenteral drugs within 90 days prior to screening | 0 | 0 |
| Participation in an investigational drug or device study within 90 days or 5 times the elimination half-life (whichever is longer) prior to screening | 0 | 0 |
| Current chronic daily treatment (continuous for >3 months) with corticosteroids (dose ≥10 mg/day methylprednisolone), excluding inhaled corticosteroids | 0 | 0 |
| Receipt of IV antibiotics for infection within 7 days prior to enrollment into the study | 0 | 0 |
| Any subject whose injection is not successfully performed | 0 | 0 |
| SC injection site other than thigh is used | 0 | 0 |
| PPAP1 | 68 | 73 |
| Excluded from PPAP1 | 4 | 1 |
| PPAP2 | 68 | 74 |
| Excluded from PPAP2 | 4 | 0 |
| TPAP1 | 68 | 73 |
| Excluded from TPAP1 | 4 | 1 |
| TPAP2 | 68 | 74 |
| Excluded from TPAP2 | 4 | 0 |

^a^Includes all randomized subjects.

^b^Includes all subjects who received at least one dose of study medication.

^c^Includes all randomized subjects who were treated and adhered to the pre-specified protocol criteria.

*AUC* area under the time–concentration curve*; BMI* body mass index*; C_max_* maximum serum concentration; *FDC* fixed-dose combination; *H*trastuzumab; *ITT* intention-to-treat; *IV* intravenous; *OBI* on-body injector; *P* pertuzumab; *PAP* per protocol PK analysis population; *P* pertuzumab; *PK* pharmacokinetic; *PPAP1* per protocol PK analysis population for pertuzumab AUC_(0–62)_; *PPAP2* per protocol PK analysis population for pertuzumab C_max_; *SC* subcutaneous; *TPAP1* per protocol PK analysis population for trastuzumab AUC_(0-62)_; *TPAP2* per protocol PK analysis population for trastuzumab C_max_

**Supplemental Table 2** Summary of Visual Analogue Scale (assessment of pain^*^) in the safety population

|  | **PH FDC SC**  **using handheld syringe**  **(*n* = 72)** | **PH FDC SC**  **using OBI**  ***(n* = 74)** |
| --- | --- | --- |
| **Time point** | **Value at visit** | **Value at visit** |
| **Prior to injection** | | |
| *n* | 72 | 74 |
| Mean (SD) | 0.68 (1.5) | 0.50 (1.5) |
| Median | 0 | 0 |
| Min–max | 0.0–8.0 | 0.0–12.0 |
| **During injection** | | |
| *n* | 72 | 74 |
| Mean (SD) | 6.9 (8.8) | 9.0 (10.3) |
| Median | 5.0 | 6.0 |
| Min–max | 0.0–62.0 | 0.0–65.0 |
| **Immediately post-injection** | | |
| *n* | 72 | 74 |
| Mean (SD) | 8.6 (12.6) | 5.0 (7.8) |
| Median | 4.5 | 2.0 |
| Min–max | 0.0–61.0 | 0.0–48.0 |
| **2 hours after injection** | | |
| *n* | 72 | 74 |
| Mean (SD) | 3.1 (5.3) | 1.6 (6.0) |
| Median | 1.0 | 0.0 |
| Min–max | 0.0–24.0 | 0.0–50.0 |

^*^Pain intensity scores assessed using the Visual Analog Scale (VAS) on a line measuring between 0 mm (‘no pain’) and 100 mm (‘unbearable pain’).

*FDC* fixed-dose combination; *H* trastuzumab; *OBI* on-body injector; *P* pertuzumab; *SC* subcutaneous; *SD* standard deviation

**Supplemental Table 3** Summary of skin irritation and sensitization reactions by timepoint in the safety population

| **Time point** | **PH FDC SC**  **using OBI**  **(*n* = 74)** |
| --- | --- |
| **Prior to injection** | |
| Total number of dermal effects respondents | 74 |
| 0 No evidence or irritation | 74 (100%) |
| 1 Minimal erythema | 0 |
| 2 Definite erythema, minimal edema or papular response | 0 |
| 3 Erythema and papules | 0 |
| 4 Definite erythema | 0 |
| 5 Erythema, edema and papules | 0 |
| 6 Vesicular eruption | 0 |
| 7 Strong reaction spreading beyond test site | 0 |
| Total number of other effects respondents | 74 |
| 0 No evidence | 74 (100%) |
| 1 Slight glazed appearance | 0 |
| 2 Marked glazing | 0 |
| 3 Glazing with peeling | 0 |
| 4 Glazing with peeling and cracking | 0 |
| 5 Glazing with fissures | 0 |
| 6 Film of dried serious exudate covering all parts of OBI site | 0 |
| 7 Small petechial eruptions or scabs | 0 |
| **After injection** |  |
| Total number of dermal effects respondents | 74 |
| 0 No evidence or irritation | 62 (83.8%) |
| 1 Minimal erythema | 12 (16.2%) |
| 2 Definite erythema, minimal edema or papular response | 0 |
| 3 Erythema and papules | 0 |
| 4 Definite erythema | 0 |
| 5 Erythema, edema and papules | 0 |
| 6 Vesicular eruption | 0 |
| 7 Strong reaction spreading beyond test site | 0 |
| Total number of other effects respondents | 74 |
| 0 No evidence | 73 (98.6%) |
| 1 Slight glazed appearance | 1 (1.4%) |
| 2 Marked glazing | 0 |
| 3 Glazing with peeling | 0 |
| 4 Glazing with peeling and cracking | 0 |
| 5 Glazing with fissures | 0 |
| 6 Film of dried serious exudate covering all parts of OBI site | 0 |
| 7 Small petechial eruptions or scabs | 0 |

*FDC* fixed-dose combination; *H* trastuzumab; *OBI* on-body injector; *P* pertuzumab; *SC* subcutaneous;

*SD* standard deviation

**Supplemental Table 4** Summary of PH FDC SC OBI assessment in the safety population

| **Question item** | **PH FDC SC OBI**  **(*n* = 74)** |
| --- | --- |
| **Ease of device attachment** | |
| Total number of respondents | 74 |
| Good | 74 (100%) |
| Acceptable | 0 |
| Poor | 0 |
| **Attachment of device during injection** | |
| Total number of respondents | 74 |
| Good | 73 (98.6%) |
| Acceptable | 1 (1.4%) |
| Poor | 0 |
| **Ease of device removal** | |
| Total number of respondents | 74 |
| Good | 67 (90.5%) |
| Acceptable | 6 (8.1%) |
| Poor | 1 (1.4%) |
| **Overall wearing comfort** | |
| Total number of respondents | 74 |
| Good | 67 (90.5%) |
| Acceptable | 7 (9.5%) |
| Poor | 0 |
| **Overall clarity of the handling instructions** | |
| Total number of respondents | 74 |
| Good | 74 (100%) |
| Acceptable | 0 |
| Poor | 0 |

*FDC* fixed-dose combination; *H* trastuzumab; *OBI* on-body injector
